# Differentiation of acute and four-week old myocardial infarct with Gd(ABE-DTTA)-enhanced CMR

**DOI:** 10.1186/1532-429X-12-22

**Published:** 2010-04-07

**Authors:** Robert Kirschner, Levente Toth, Akos Varga-Szemes, Tamas Simor, Pal Suranyi, Pal Kiss, Balazs Ruzsics, Attila Toth, Robert Baker, Brigitta C Brott, Silvio Litovsky, Ada Elgavish, Gabriel A Elgavish

**Affiliations:** 1 Department of Biochemistry and Molecular Genetics, University of Alabama at Birmingham, MCLM 556, Birmingham, AL 35294-0005, USA; 2 Division of Cardiovascular Disease, Department of Medicine, University of Alabama at Birmingham, Birmingham, Alabama, USA; 3 Division of Clinical Immunology and Rheumatology, Department of Medicine, University of Alabama at Birmingham, Birmingham, Alabama, USA; 4 Department of Anatomical Pathology, University of Alabama at Birmingham, Birmingham, Alabama, USA; 5 Animal Resources Program, University of Alabama at Birmingham, Birmingham, Alabama, USA; 6 Heart Institute, Faculty of Medicine, University of Pecs, Hungary; 7 Elgavish Paramagnetics Inc., Birmingham, Alabama, USA

## Abstract

**Background:**

Standard extracellular cardiovascular magnetic resonance (CMR) contrast agents (CA) do not provide differentiation between acute and older myocardial infarcts (MI). The purpose of this study was to develop a method for differentiation between acute and older myocardial infarct using myocardial late-enhancement (LE) CMR by a new, low molecular weight contrast agent.

Dogs (n = 6) were studied in a closed-chest, reperfused, double myocardial infarct model. Myocardial infarcts were generated by occluding the Left Anterior Descending (LAD) coronary artery with an angioplasty balloon for 180 min, and four weeks later occluding the Left Circumflex (LCx) coronary artery for 180 min. LE images were obtained on day 3 and day 4 after second myocardial infarct, using Gd(DTPA) (standard extracellular contrast agent) and Gd(ABE-DTTA) (new, low molecular weight contrast agent), respectively. Triphenyltetrazolium chloride (TTC) histomorphometry validated existence and location of infarcts. Hematoxylin-eosin and Masson's trichrome staining provided histologic evaluation of infarcts.

**Results:**

Gd(ABE-DTTA) or Gd(DTPA) highlighted the acute infarct, whereas the four-week old infarct was visualized by Gd(DTPA), but not by Gd(ABE-DTTA). With Gd(ABE-DTTA), the mean ± SD signal intensity enhancement (SIE) was 366 ± 166% and 24 ± 59% in the acute infarct and the four-week old infarct, respectively (P < 0.05). The latter did not differ significantly from signal intensity in healthy myocardium (P = NS). Gd(DTPA) produced signal intensity enhancements which were similar in acute (431 ± 124%) and four-week old infarcts (400 ± 124%, P = NS), and not statistically different from the Gd(ABE-DTTA)-induced SIE in acute infarct. The existence and localization of both infarcts were confirmed by triphenyltetrazolium chloride (TTC). Histologic evaluation demonstrated coagulation necrosis, inflammation, and multiple foci of calcification in the four day old infarct, while the late subacute infarct showed granulation tissue and early collagen deposition.

**Conclusions:**

Late enhancement CMR with separate administrations of standard extracellular contrast agent, Gd(DTPA), and the new low molecular weight contrast agent, Gd(ABE-DTTA), differentiates between acute and late subacute infarct in a reperfused, double infarct, canine model.

## Background

Reinfarction occurs in 7-8% of cardiac patients with previous MI [1,2]. In a recent meta-analysis [3], with 6921 patients with MI treated primarily with balloon angioplasty with or without stenting, the rate of reinfarction was ~3% in the first year. Differentiation between acute and older MIs is of great importance in clinical decision-making. Wall motion abnormalities detected with echocardiography, computed tomography (CT), or cardiovascular magnetic resonance (CMR) are not restricted to acute events. Also, regardless of age of MI, radioactive tracers are not taken up by non-viable myocardial cells imaging, and therefore both recent and long-standing MI appears as a fixed defect. Not even late enhancement (LE) CMR with standard extracellular contrast agents (CA) like Gadolinium-DTPA (Gadolinium-Diethylenetriaminepentaacetic acid) differentiates by age of infarct [4,5].

We have developed a family of CAs for CMR diagnosis of ischemic heart disease (IHD) [6-9]. Among these, Gd(ABE-DTTA) is optimal for cardiovascular purposes. Gd(ABE-DTTA), which is still under investigation, is the Gadolinium complex of N-(2-butyryloxyethyl)-N'-(2-ethyloxy-ethyl)-N, N'-bis [N", N"-bis(carboxymethyl)acetamido]-1,2-ethanediamine. This low molecular weight (764 Dalton) agent's clearance from the blood has a kinetics similar to that of blood pool contrast agents, although it also displays partly extracellular characteristics [10]. It demonstrates high affinity for acute MI [10]. The acutely infarcted tissue takes up Gd(ABE-DTTA) within a longer period of time than it does purely extracellular contrast agents. The maximum concentration of the agent in the acutely infarcted tissue shows up at 48 hr, although it is not significantly different from that which is already achieved at 24 hr, and the contrast remains detectable in the infarct up to 12 days [10]. The agent causes no deleterious physiological effects, and a previous study has demonstrated the short- and long term safety of its usage [11].

Gd(ABE-DTTA) has been successfully used for continuous detection of myocardial ischemia during 30 min of left anterior descending coronary artery (LAD) occlusion [12,13]. The suitability of Gd(ABE-DTTA) for accurate quantification of acute MI has also been demonstrated [14,15].

In this study we have shown that Gd(ABE-DTTA) induces a LE effect in acute, but not in late subacute MI.

## Methods

### Gd(ABE-DTTA) sample preparation

Gd(ABE-DTTA) was synthesized, and samples were prepared, as described by Saab et al. [8]. To guarantee consistent quality of agent before administration the in vitro relaxivity was measured for every sample [8]. Each animal received Gd(ABE-DTTA) at the dose of 0.05 mmol/kg, the in vitro relaxation enhancement of which was equivalent to that of Gd-DTPA at its conventional dose (0.2 mmol/kg) used for LE-CMR.

### Study design

**Figure 1:**
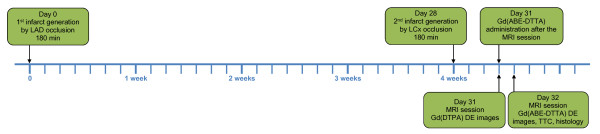
**Timeline of the Study Protocol - Double Infarct Model**. Day 0 - MI generation in LAD-supply area. Day 28 - another MI generation in LCx- supply area. Day 31 (3 days after the 2^nd ^MI) - CMR session: obtaining LE images with Gd(DTPA), followed by Gd(ABE-DTTA) administration. Day 32 (4 days after the 2^nd ^MI) - CMR session: obtaining LE images with Gd(ABE-DTTA), followed by TTC, and histology.

Animals were studied with a closed-chest, reperfused, double MI protocol described below. The smallest number of animals (n = 6) that still achieved statistical significance was used. MIs were generated in the LAD coronary artery territory and four weeks later in that of the left circumflex coronary artery (LCx) (Figure 1.). To avoid a confounding, simultaneous action of the two contrast agents, two separate CMR sessions were carried out 3 and 4 days after the generation of the second MI, separately using Gd(DTPA) and Gd(ABE-DTTA) in these two sessions, respectively.

### Surgical procedure

Animal protocol was approved by the University of Alabama at Birmingham IACUC in full compliance with the 'Guidelines for the Care and use for Laboratory Animals' (NIH). Six male hounds (18-20 kg) were used. Twelve hours prior to procedure food was taken away and 325 mg Aspirin given. Dogs were anesthetized with a Ketamine (5.0 mg/kg) and Diazepam(0.5 mg/kg) mixture, intubated, and connected to a Hallowell EMC Model 2000 respirator (Pittsfield, MA, USA) operated with a tidal volume of 400 ml at a rate of 16 BPM. Anesthesia was maintained by continuous Isoflurane (2.5-3 volume %), and repeated Fentanyl (50-100 ug I.V. every 30 minutes), administration. Heart rate and blood oxygen saturation were monitored using a pulse-oxymeter placed on the animal's tongue. ECG electrodes were placed on the chest to record electrophysiological signs of myocardial ischemia and arrhythmias. The left femoral artery was separated surgically and an arterial sheath (6-8 French) was inserted. An I.V. line was placed to administer infusion and drugs. Heparin (100 IU/kg) was given intravenously to maintain the activated clotting time (ACT) above 300 seconds. A properly sized 2-3 mm angioplasty balloon was introduced under fluoroscopic guidance into the LAD (1^st ^infarct) or the LCx (2^nd ^infarct) and inflated for 180 minutes to create MI. Thereafter, the balloon was deflated to restore coronary circulation. Coronary angiography confirmed the reperfusion after balloon deflation.

On days 3 and 4 after the second infarction, animals were re-anesthetized as described above and CMR studies performed. Animals were then sacrificed, hearts excised and embedded in agar. The agar block was cut perpendicular to the long axis with a commercial meat slicer into 5 mm sections starting from the apex. 2,3,5-triphenyltetrazolium chloride (TTC) was dissolved in physiological saline to obtain 2% TTC solution. Slices were immersed in it at 37°C for 15 min and then rinsed with physiological saline. All TTC-stained slices were photographed with a high resolution digital camera. TTC-stained slices were used to validate the existence and location of infarcts.

### Magnetic Resonance Imaging

A 1.5T GE Signa-Horizon CV/i scanner (Milwaukee, WI, USA) was used. A cardiac phased-array coil and ECG gating were employed. Breath-hold was performed at end-expiration. A 180°-prepared, segmented, inversion-recovery fast gradient-echo pulse was used with: Field of View (FOV) 30 cm, Echo Time (TE) 3.32 ms, Repetition Time (TR) two cardiac cycles (1100-1600 ms), slice thickness 10 mm. The Inversion Time (TI) was optimized to null the signal normal myocardium. Conventional cardiac angulation planes were set and short axis slices covering the entire left ventricle (LV) obtained (six slices per heart).

In the first CMR session, a 0.2 mmol/kg Gd(DTPA) (Magnevist, Schering, Kenilworth, NJ) bolus was administered intravenously. LE images were acquired 15-20 min thereafter. Gd(ABE-DTTA) was given intravenously at the end of the first CMR session. In the second CMR session, 24 h after Gd(ABE-DTTA) administration, LE images were similarly obtained.

### Histology

Post mortem tissue samples from the infarct and the peri-infarct regions were examined by histopathology. The samples were fixed in 10% formalin, embedded in paraffin, and sectioned at 5 μm thickness. Hematoxylin-eosin and Masson's trichrome staining was performed.

### Image analysis

The existence of both acute and four-week old infarcts was validated and their anatomical localization determined by analyzing the TTC images. CMR Dicom images were imported as image sequences with the use of ImageJ (Wayne Rasband, NIH). The endo- and epicardial contours of the LV muscle were traced manually and this circumscribed area was further analyzed. Based on the apicobasal localization and anatomical landmarks (LV and papillary muscle shape, and the position of the anterior and posterior interventricular grooves), CMR images acquired in the presence of the two different contrast agents, as well as the TTC slices, were matched. All CMR slices that contained a MI according to the corresponding TTC slices were categorized into four groups by anatomical localization of the infarct to either the four-week old or to the acute category, and by the contrast agent given.

To separate the acute and the four-week old infarcts for the analysis, the images of slices containing both types of MI were partitioned into two images, each reflecting one half of the tomographic slice. The partition was done, with ImageJ, along a straight line starting at the posterior interventricular groove (0° on the LV circumference), through the center point of the LV slice, ending at the 180° point on the LV circumference in the anterolateral region. Thus four groups of images were obtained for analysis: Gd(DTPA)_acute_, Gd(DTPA)_4 week_, Gd(ABE-DTTA)_acute_, Gd(ABE-DTTA)_4 week_, to which two control groups, (Gd(DTPA)_normal_, Gd(ABE-DTTA)_normal_), i.e. normal myocardium with each of the two agents, have been added, bringing the number of data groups for analysis to six.

To avoid observer bias, instead of manual contouring of the infarct and the healthy myocardial regions, a pixel-by-pixel analysis was performed. The pixel-by-pixel SI histogram of every CMR image, segmented in the above manner, was generated with ImageJ and these histograms were used for further analysis. First, the mean SI ± SD of healthy myocardium was determined by exporting the histograms to Origin 7.0 (OriginLab Corporation, Massachusetts, USA), and employing Gaussian curve fitting on each using the Levenberg-Marquardt algorithm [16]. In agreement with a previous publication [17], pixels with SI above the mean + 6 SD of the normal myocardium were regarded as enhanced pixels, i.e. pixels of the infarct. The mean SI of these enhanced pixels was calculated from this set of pixels in each image. If no pixels above the threshold were found, the mean signal intensity of the infarct was concluded to be equal to that of healthy myocardium. The mean SIE in each pixel was computed by [18]:

where SI_i _and SI_n _are the mean signal intensity in infarct and normal myocardium, respectively.

### Statistical analysis

Results are reported as mean ± SD. Statistical analysis was carried out by SigmaStat (version 2.03; SPSS Inc, Chicago, IL, USA). Two-way repeated measures analysis of variance was used to compare the SIE values among the six experimental groups. Normal distribution and equality of variances were tested. Although the test of normality failed, due to the equality of variances, the equality of group sizes, and the high power of the performed test (0.985 with α = 0.05), the assumption of the F test in the two-way ANOVA with repeated measures was not violated [19]. Since an overall significance (P < 0.05) was established for rejecting the null hypothesis that the six groups are not different, pairwise differences between the groups were assessed by using the Holm-Sidak method of adjustment for multiple comparisons.

## Results

**Figure 2:**
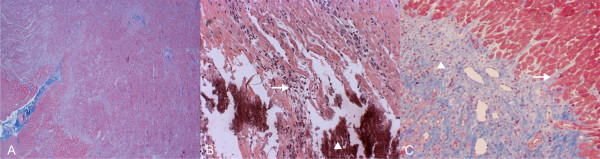
**Double Infarct Histology**. Histologic section of a dog LV 32 days following the first (LAD), and 4 days after second (LCx), MI. (A) Acute (LCx) MI with coagulation necrosis, inflammation and multiple foci of calcification (40×, Masson's trichrome). (B) Same area at higher magnification. Dying myocardial fibers (white arrow) associated with inflammatory cells, Calcium precipitates (white arrowhead) (100×). (C) Late subacute (LAD) MI in the same heart with granulation tissue (white arrowhead) and early collagen deposition, interdigitating (white arrow) with viable myofibers (40×, Masson's trichrome).

**Figure 3:**
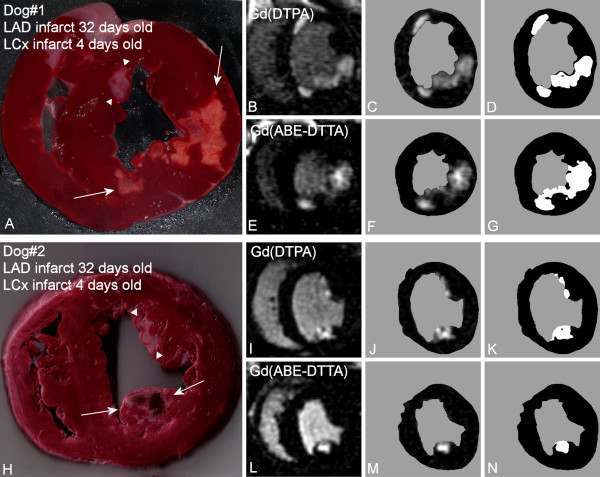
**Differentiation between Acute vs. Subacute Myocardial Infarctions**. A: TTC stained photograph of a canine (Dog#1) transversal LV slice 32 days following the first (LAD) and 4 days after second (LCx) infarct. White arrows point to the acute infarct located in the posterior and postero-lateral segments (LCx-supply area). Subacute infarct (white arrowheads) is seen on the border of the anteroseptal and anterior segment (LAD-supply area). B: Corresponding LE CMR image taken in the presence of Gd(DTPA). This CA does not differentiate between the acute and the subacute infarcts. C: Same as B, after the endo- and epicardial contours of the LV muscle had been traced manually D: Image in C thresholded at normal+6SD intensity. E: Same CMR image as in B, taken in the presence of Gd(ABE-DTTA) one day following B. Only the posterior and postero-lateral segments, i.e. the acute infarct, show LE. The subacute infarct is not highlighted by this agent, thereby differentiating between acute and subacute infarcts. F: Same as E, after the endo- and epicardial contours of the LV muscle had been traced manually. G: Image F thresholded at normal+6SD intensity. H: TTC photograph of *another *canine (Dog#2) LV slice 32 days following first (LAD) and 4 days after second (LCx) infarct. White arrows point to the acute (hemorrhagic) infarct in the posteromedial papillary muscle (LCx-supply area). Subacute infarct (white arrowheads) is seen predominantly in the anterolateral papillary muscle (LAD-supply area). I: Corresponding LE image with Gd(DTPA). J: Same as I, after the contours of the LV muscle had been traced K: Image J thresholded at normal+6SD L: Same CMR image as in I, taken in the presence of Gd(ABE-DTTA) one day following I. Only the acute infarct shows LE. The subacute infarct is not highlighted by this agent. M: Same as L, after the contours had been traced N: Image M thresholded at normal+6SD.

**Figure 4:**
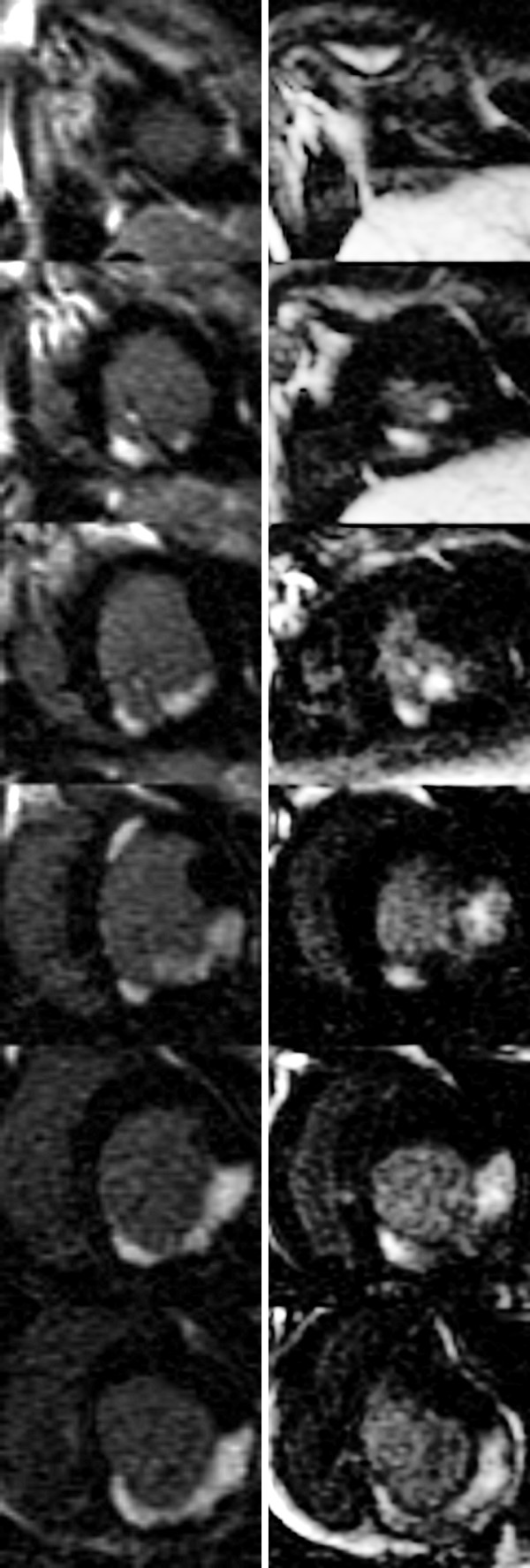
**Short Axis LE CMR Image Set -Dog#1**. All base-apex slices of dog#1 (see also Figure 3). Left column - LE images with Gd(DTPA). Right column - LE images with Gd(ABE-DTTA).

**Figure 5:**
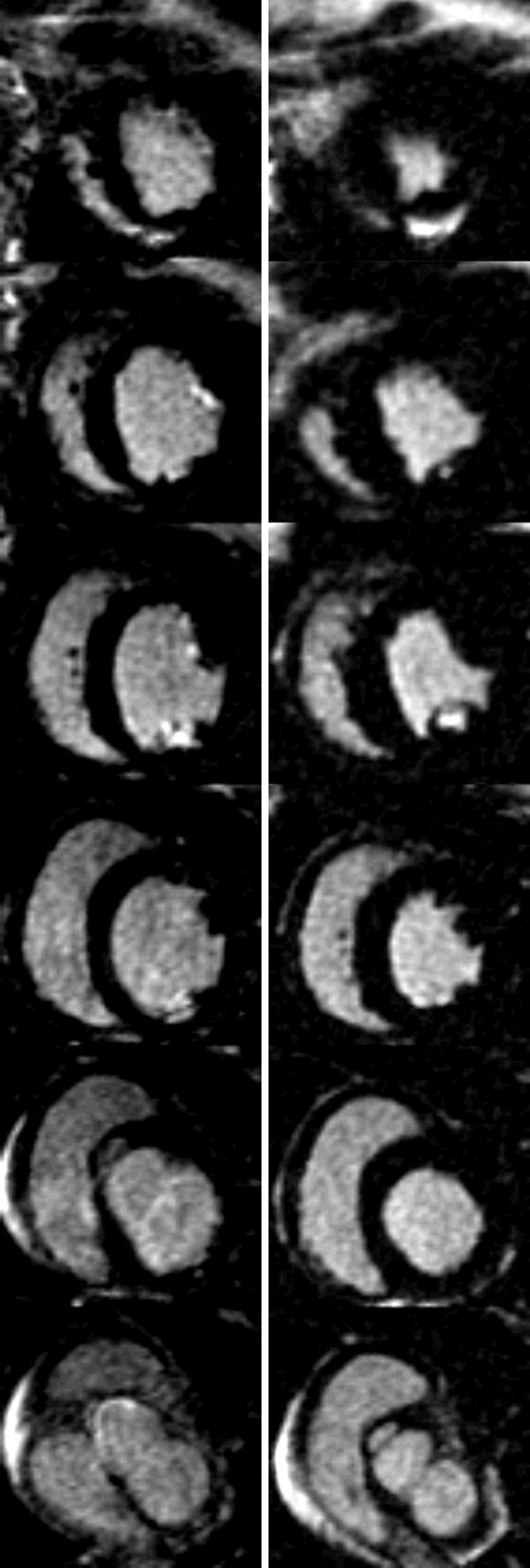
**Short Axis LE CMR Image Set -Dog#2**. All base-apex slices of dog#2 (see also Figure 3). Left column - LE images with Gd(DTPA). Right column - LE images with Gd(ABE-DTTA).

Both the acute (LCx) and four-week old (LAD) infarcts were visible in Gd(DTPA)-enhanced LE images of all six dogs. The existence and localization of recent and four-week old infarct were confirmed by TTC. Histologic evaluation confirmed acute infarcts with coagulation necrosis, inflammation (mostly mononuclear), and multiple foci of calcification in the 4 days old infarct areas (Figure 2.). Healing with granulation tissue and early collagen deposition, and small areas of interstitial fibrosis adjacent to the late subacute infarct were seen in the four-week old infarct areas. Gd(ABE-DTTA) did not induce SIE in the subacute (LAD) infarcts, while the acute (LCx) infarcts were clearly visible on LE images of all six animals in the presence of this CA (see examples in Figure 3, 4, and 5).

**Figure 6:**
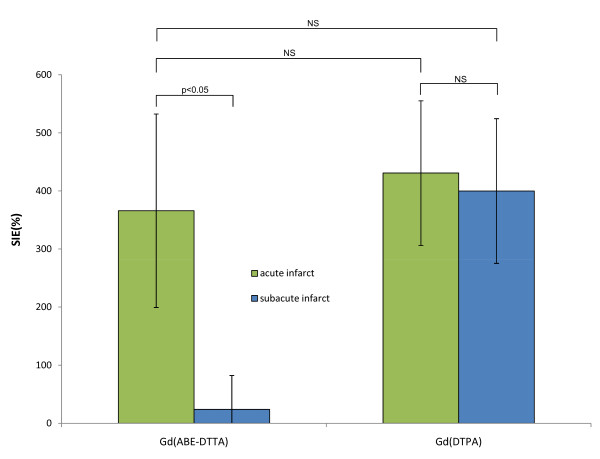
**Comparison of SIE Values**. Mean (n = 6) Signal Intensity Enhancement (%) induced by Gd(ABE-DTTA) or Gd(DTPA) in areas of acute (green bars) and subacute infarcts (blue bars).

Mean ± SD SIE values are shown in Table 1 and Figure 6. With Gd(ABE-DTTA), the mean SIE in the areas with acute infarct was 366 ± 167%, whereas in areas of four-week old infarcts it was only 24 ± 59%. The difference is statistically significant (P < 0.05). The mean SIE in four-week old infarct areas with Gd(ABE-DTTA) did not differ significantly from SIE of healthy myocardium (P = NS). In contradistinction, Gd(DTPA) produced similar mean SIEs in acute (430 ± 124%) and four-week old infarcts (400 ± 124%, P = NS). Furthermore, the mean SIE values of neither acute nor four-week old infarcts enhanced with Gd(DTPA) were statistically different from mean SIE of acute infarct areas enhanced with Gd(ABE-DTTA). These data show that Gd(ABE-DTTA) differentiates between acute and 4 week-old infarcts, and induces approximately the same SIE in acute infarcts as Gd(DTPA) does.

**Table 1:** Contrast Induced by the Two Agents in Acute versus Subacute Infarcts

	Gd(ABE-DTTA)		Gd(DTPA)	
		
Parameter	Acute infarct	Subacute infarct	Acute infarct	Subacute infarct
SIE (%)*	366 ± 166	24 ± 59	431 ± 124	400 ± 124
P value† vs. normal myocardium	P < 0.05	NS	P < 0.05	P < 0.05
P value vs. Gd(ABE-DTTA)_subacute_	P < 0.05		P < 0.05	P < 0.05
P value vs. Gd(ABE-DTTA)_acute_			NS	NS
P value vs. Gd(DTPA)_acute_				NS

*Mean ± SD (n = 6) signal intensity enhancement (in percent values) by Gd(ABE-DTTA) or Gd(DTPA) in the territory of acute or subacute occlusions

†P values pertain to pairwise comparisons by the Holm-Sidak method of the different subgroups.

## Discussion

Gd(ABE-DTTA) was capable of differentiating between acute and four-week old infarcts as no LE effect was seen in the latter while one is clearly observable in the former. Acute MIs can be seen on the LE-CMR images enhanced with either Gd(DTPA) or Gd(ABE-DTTA). Older MIs are visible only by Gd(DTPA). Our general observations show in dogs that the agent's affinity to infarcted myocardial tissue disappears between days 10 and 14 following acute myocardial infarction. The question of the detailed kinetics of effect disappearance is currently under investigation.

### Magnetic resonance imaging for differentiation between new and longstanding MI

Saeed et al. [20] have recently published similar observations with an intravascular, high molecular weight contrast agent, P792, Vistarem (Guerbet Group, Paris, France). They studied one infarct at two different time-points in a single MI model. The intravascular agent produced a LE in the acute, 3 day old infarct but not in the later, chronic phase, at 8 weeks, after infarction. The authors have also proven the superiority of their method over the T2-weighted (T2w) CMR technique (also see below) for the distinction between acute and chronic myocardial infarct.

Other methods for the differentiation between new and longstanding MIs have also been published. In a recent publication Hillenbrand et al. [21] reported a method with the combination of Gd(DTPA)-enhanced ^1^H MR and ^23^Na MR. Due to granulation tissue infiltrates and collagen deposition, the ^23^Na MR signal intensity in the MI area showed a significant decrease during infarct healing. Another approach by Abdel-Aty et al. [22] combined Gd(DTPA)-induced LE with T2-weighted (T2w) CMR. The distinction was based on an elevated T2w signal intensity due to edema which is located selectively in the newly infarcted tissue [22]. The specificity of this method could be limited by the fact, however, that sometimes, especially in the case of ongoing residual ischemia, sustained postinfarction edema can be detected for up to one year after MI [23]. Kim et al. [24] used contrast enhanced steady state free precession (SSFP) CMR for the distinction between recent and chronic MIs. The mean signal intensity of acute infarct areas was elevated during SSFP CMR, two minutes after Gd(DTPA) administration. Chronic infarct regions, however, showed signal intensities similar to that of normal myocardium. The exact mechanism of the LE phenomenon is not known to date even for standard extracellular agents. Increased volume of distribution, due to sarcolemmal membrane rupture in acute infarct, and low grade of cellularity with expanded interstitial collagen matrix of chronic scar tissue, have been suggested as potential mechanisms for such agents [4,5,25-27].

### Histological basis of the CMR observation

The evolution of MI is a complex pathohistological process [28]. Several hypotheses can be brought up to explain the selective accumulation of Gd(ABE-DTTA) into acute MIs. The first is linked to the partial intravascular nature of Gd(ABE-DTTA) [10]. Microvascular damage in an acute infarct [28] could lead to increased microvascular permeability towards CAs with intravascular behavior [29,30], while the remodeled microvessels in healing infarcts [20,31] would reduce such permeability. Having partial intravascular characteristics [10], the above mentioned process could increase the wash-in constant of Gd(ABE-DTTA) in acute infarcts, and decrease it in four-week old infarcts. A second hypothesis may be attributable to a possible necrosis-avidity of Gd(ABE-DTTA). There may be binding sites for the CA among the different elements of acute necrotic tissue such as subcellular compartments (ruptured membrane, cytosol, mitochondria), calcium precipitates [28,32], or ingredients of acute inflammatory reactions, persisting selectively in acutely infarcted tissue. Thus the progressive, persistent [10] accumulation in acutely infarcted tissue of Gd(ABE-DTTA) may be partly due to its partial lipophilic nature [8], whereby lipids derived from the above mentioned cellular components may trap this CA.

### Study limitations

Our study has some limitations. The LE images with the two different CAs were performed on two consecutive days. Thus, small differences in selection of the planes of the short-axis images between the two CMR sessions cannot be completely excluded. These differences, however, would not impair the outcome of our studies.

On day 31, Gd(ABE-DTTA) was administered at the end of the first CMR session. It brings up the possibility, as Gd(DTPA) (in spite of its short half life time) might not have been fully cleared from the body yet, that the two contrast agents could potentially interact with each other. There is no chemical basis, however, for the assumption of interaction, and that it could have influenced the late enhancement phenomenon in the second CMR session, i.e. 24 hours later. Publications [10,14] already demonstrated the ability of Gd(ABE-DTTA) to detect acute infarcts. These publications used a protocol where Gd(ABE-DTTA) was administered alone.

Gd(ABE-DTTA) needs to be administered 24 hours before CMR imaging, and this introduces an inconvenience in a clinical setting. It may not be convenient (the cardiology ward and radiology are not necessarily close), nor practical for answering the urgent clinical question in a timely fashion. This mode of contrast agent administration, however, is not unknown in the practice of nuclear cardiology. For example, there is a protocol for the assessing of myocardial viability with rest redistribution ^201^Thallium SPECT (Single Photon Emission Computed Tomography), where repeated delayed imaging is employed 3-4 h or 24 h following the administration of the radiotracer [33].

The slow clearance of Gd(ABE-DTTA) from the body suggests that the time available for undesired physiological effects, such as dissociation of Gd from the chelate, may be considerably longer than is the case for agents currently approved for human use. It is noteworthy, however, that Gd(ABE-DTTA)'s short- and long term physiological safety has been reported [11].

## Conclusions

In summary, we have shown that LE-CMR with separate administrations of Gd(DTPA) and Gd(ABE-DTTA) differentiates between acute and four-week old MIs in a reperfused, double infarct, canine model.

## Competing interests

Dr. Kirschner and Dr. Varga-Szemes are employees, Drs. A. Toth, L. Toth, P. Kiss, P. Suranyi and B. Ruzsics were employees, and Drs. A. Elgavish and G. Elgavish are officers of Elgavish Paramagnetics Inc.

## Authors' contributions

All authors participated in design and execution of experiments, analysis and interpretation of data, drafting and critical evaluation of manuscript. All authors have read and approved the manuscript.

## References

[B1] Udvarhelyi IS Gatsonis C Epstein AM Pashos CL Newhouse JP McNeil BJ Acute myocardial infarction in the Medicare population. Process of care and clinical outcomes JAMA 1992 268 2530 2536 10.1001/jama.268.18.2530 1404820

[B2] Elhendy A Schinkel AFL van Domburg RT Bax JJ Poldermans D Differential prognostic significance of peri-infarction versus remote myocardial ischemia on stress technetium-99 m sestamibi tomography in patients with healed myocardial infarction The American Journal of Cardiology 2004 94 289 293 10.1016/j.amjcard.2004.04.021 15276090

[B3] De Luca G Suryapranata H Stone G Antoniucci D Biondi-Zoccai G Kastrati A Chiariello M Marino P Coronary stenting versus balloon angioplasty for acute myocardial infarction: a meta-regression analysis of randomized trials Int J Cardiol 2008 126 37 44 10.1016/j.ijcard.2007.03.112 17544528

[B4] Mahrholdt H Wagner A Holly TA Elliott MD Bonow RO Kim RJ Judd RM Reproducibility of Chronic Infarct Size Measurement by Contrast-Enhanced Magnetic Resonance Imaging Circulation 2002 106 2322 2327 10.1161/01.CIR.0000036368.63317.1C 12403661

[B5] Choi KM Kim RJ Gubernikoff G Vargas JD Parker M Judd RM Transmural extent of acute myocardial infarction predicts long-term improvement in contractile function Circulation 2001 104 1101 1107 10.1161/hc3501.096798 11535563

[B6] Kim SK Pohost GM Elgavish GA Fatty-acyl iminopolycarboxylates: lipophilic bifunctional contrast agents for NMR imaging Magn Reson Med 1991 22 57 67 10.1002/mrm.1910220107 1798395

[B7] Chu WJ Simor T Elgavish GA In vivo characterization of Gd(BME-DTTA), a myocardial MRI contrast agent: tissue distribution of its MRI intensity enhancement, and its effect on heart function NMR in Biomedicine 1997 10 87 92 10.1002/(SICI)1099-1492(199704)10:2<87::AID-NBM438>3.0.CO;2-T 9267866

[B8] Saab-Ismail NH Simor T Gaszner B Lorand T Szollosy M Elgavish GA Synthesis and in vivo evaluation of new contrast agents for cardiac MRI J Med Chem 1999 42 2852 2861 10.1021/jm980454v 10425094

[B9] Simor T Chu WJ Johnson L Safranko A Doyle M Pohost GM Elgavish GA In vivo MRI visualization of acute myocardial ischemia and reperfusion in ferrets by the persistent action of the contrast agent Gd (BME-DTTA) Circulation 1995 92 3549 3559 8521578 10.1161/01.cir.92.12.3549

[B10] Suranyi P Kiss P Ruzsics B Brott BC Simor T Elgavish A Baker RA Saab-Ismail NH Elgavish GA In vivo myocardial tissue kinetics of Gd(ABE-DTTA), a tissue-persistent contrast agent Magn Reson Med 2007 58 55 64 10.1002/mrm.21249 17659616

[B11] Ruzsics B Surányi P Kiss P Brott B Elgavish A Saab-Ismail N Simor T Elgavish G Gd(ABE-DTTA), a Novel Contrast Agent, at the MRI-Effective Dose Shows Absence of Deleterious Physiological Effects in Dogs Pharmacology 2006 77 188 194 10.1159/000094864 16877874

[B12] Simor T Gaszner B Oshinski JN Waldrop SM Pettigrew RI Horvath IG Hild G Elgavish GA Gd(ABE-DTTA)-enhanced cardiac MRI for the diagnosis of ischemic events in the heart J Magn Reson Imaging 2005 21 536 545 10.1002/jmri.20326 15834916

[B13] Kiss P Suranyi P Simor T Saab-Ismail NH Elgavish A Hejjel L Elgavish GA In vivo R1-enhancement mapping of canine myocardium using ceMRI with Gd(ABE-DTTA) in an acute ischemia-reperfusion model J Magn Reson Imaging 2006 24 571 579 10.1002/jmri.20661 16892191

[B14] Suranyi P Kiss P Brott BC Simor T Elgavish A Ruzsics B Saab-Ismail NH Elgavish GA Percent infarct mapping: an R1-map-based CE-MRI method for determining myocardial viability distribution Magn Reson Med 2006 56 535 545 10.1002/mrm.20979 16892184

[B15] Ruzsics B Suranyi P Kiss P Brott BC Elgavish A Simor T Elgavish GA Head-to-head comparison between delayed enhancement and percent infarct mapping for assessment of myocardial infarct size in a canine model J Magn Reson Imaging 2008 28 1386 1392 10.1002/jmri.21571 19025946

[B16] Donald WM An Algorithm for Least-Squares Estimation of Nonlinear Parameters SIAM Journal on Applied Mathematics 1963 11 431 441 10.1137/0111030

[B17] Beek AM Bondarenko O Afsharzada F van Rossum AC Quantification of late gadolinium enhanced CMR in viability assessment in chronic ischemic heart disease: a comparison to functional outcome J Cardiovasc Magn Reson 2009 11 6 10.1186/1532-429X-11-6 19272147 PMC2657135

[B18] Simonetti OP Kim RJ Fieno DS Hillenbrand HB Wu E Bundy JM Finn JP Judd RM An improved MR imaging technique for the visualization of myocardial infarction Radiology 2001 218 215 223 11152805 10.1148/radiology.218.1.r01ja50215

[B19] Witte RS Witte JS Statistics 1997 5 Fort Worth: Harcourt Brace College Publishers

[B20] Saeed M Weber O Lee R Do L Martin A Saloner D Ursell P Robert P Corot C Higgins CB Discrimination of myocardial acute and chronic (scar) infarctions on delayed contrast enhanced magnetic resonance imaging with intravascular magnetic resonance contrast media J Am Coll Cardiol 2006 48 1961 1968 10.1016/j.jacc.2006.03.071 17112985

[B21] Hillenbrand HB Becker LC Kharrazian R Hu K Rochitte CE Kim RJ Chen EL Ertl G Hruban RH Lima JA 23Na MRI combined with contrast-enhanced 1H MRI provides in vivo characterization of infarct healing Magn Reson Med 2005 53 843 850 10.1002/mrm.20417 15799052

[B22] Abdel-Aty H Zagrosek A Schulz-Menger J Taylor AJ Messroghli D Kumar A Gross M Dietz R Friedrich MG Delayed Enhancement and T2-Weighted Cardiovascular Magnetic Resonance Imaging Differentiate Acute From Chronic Myocardial Infarction Circulation 2004 109 2411 2416 10.1161/01.CIR.0000127428.10985.C6 15123531

[B23] Nilsson JC Nielsen G Groenning BA Fritz-Hansen T Sondergaard L Jensen GB Larsson HB Sustained postinfarction myocardial oedema in humans visualised by magnetic resonance imaging Heart 2001 85 639 642 10.1136/heart.85.6.639 11359743 PMC1729755

[B24] Kim KA Seo JB Do KH Heo JN Lee YK Song JW Lee JS Song KS Lim TH Differentiation of recently infarcted myocardium from chronic myocardial scar: the value of contrast-enhanced SSFP-based cine MR imaging Korean J Radiol 2006 7 14 19 10.3348/kjr.2006.7.1.14 16549951 PMC2667572

[B25] Kim RJ Fieno DS Parrish TB Harris K Chen EL Simonetti O Bundy J Finn JP Klocke FJ Judd RM Relationship of MRI delayed contrast enhancement to irreversible injury, infarct age, and contractile function Circulation 1999 100 1992 2002 10556226 10.1161/01.cir.100.19.1992

[B26] Kim RJ Chen EL Lima JA Judd RM Myocardial Gd-DTPA Kinetics Determine MRI Contrast Enhancement and Reflect the Extent and Severity of Myocardial Injury After Acute Reperfused Infarction Circulation 1996 94 3318 3326 8989146 10.1161/01.cir.94.12.3318

[B27] Fieno DS Kim RJ Chen EL Lomasney JW Klocke FJ Judd RM Contrast-enhanced magnetic resonance imaging of myocardium at risk: distinction between reversible and irreversible injury throughout infarct healing J Am Coll Cardiol 2000 36 1985 1991 10.1016/S0735-1097(00)00958-X 11092675

[B28] Fishbein MC Maclean D Maroko PR The histopathologic evolution of myocardial infarction Chest 1978 73 843 849 10.1378/chest.73.6.843 657859

[B29] Krombach GA Wendland MF Higgins CB Saeed M MR Imaging of Spatial Extent of Microvascular Injury in Reperfused Ischemically Injured Rat Myocardium: Value of Blood Pool Ultrasmall Superparamagnetic Particles of Iron Oxide Radiology 2002 225 479 486 10.1148/radiol.2252011512 12409583

[B30] Saeed M van Dijke CF Mann JS Wendland MF Rosenau W Higgins CB Brasch RC Histologic confirmation of microvascular hyperpermeability to macromolecular MR contrast medium in reperfused myocardial infarction J Magn Reson Imaging 1998 8 561 567 10.1002/jmri.1880080308 9626869

[B31] Ren G Michael LH Entman ML Frangogiannis NG Morphological characteristics of the microvasculature in healing myocardial infarcts J Histochem Cytochem 2002 50 71 79 11748296 10.1177/002215540205000108

[B32] Marchal G Ni Y Herijgers P Flameng W Petre C Bosmans H Yu J Ebert W Hilger C Pfefferer D Semmler W Baert A Paramagnetic metalloporphyrins: infarct avid contrast agents for diagnosis of acute myocardial infarction by MRI Eur Radiol 1996 6 2 8 10.1007/BF00619942 8797942

[B33] Vesely MR Dilsizian V Nuclear Cardiac Stress Testing in the Era of Molecular Medicine J Nucl Med 2008 49 399 413 10.2967/jnumed.107.033530 18322120

